# Integrated analysis of transcriptomics and metabolomics of garden asparagus *(Asparagus officinalis L.)* under drought stress

**DOI:** 10.1186/s12870-024-05286-z

**Published:** 2024-06-15

**Authors:** Xuhong Zhang, Changzhi Han, Yubo Wang, Tao Liu, Yuqin Liang, Yanpo Cao

**Affiliations:** 1 Institute of Cash Crops, Hebei Academy of Agriculture and Forestry Sciences, Shijiazhuang, China; 2 Shijiazhuang Landscape Management and Protection Center, Shijiazhuang, China; 3 College of Biodiversity Conservation, Southwest Forestry University, Kunming, China; 4 School of Landscape and Ecological Engineering, Hebei University of Engineering, Handan, China

**Keywords:** Drought stress, Transcriptome, Metabolome, Metabolic pathways, Asparagus

## Abstract

**Background:**

Drought is a leading environmental factor affecting plant growth. To explore the drought tolerance mechanism of asparagus, this study analyzed the responses of two asparagus varieties, namely, ‘Jilv3’ (drought tolerant) and ‘Pacific Early’ (drought sensitive), to drought stress using metabolomics and transcriptomics.

**Results:**

In total, 2,567 and 7,187 differentially expressed genes (DEGs) were identified in ‘Pacific Early’ and ‘Jilv3’, respectively, by comparing the transcriptome expression patterns between the normal watering treatment and the drought stress treatment. These DEGs were significantly enriched in the amino acid biosynthesis, carbon metabolism, phenylpropanoid biosynthesis, and plant hormone signal transduction pathways. In ‘Jilv3’, DEGs were also enriched in the following energy metabolism-related pathways: citrate cycle (TCA cycle), glycolysis/gluconeogenesis, and pyruvate metabolism. This study also identified 112 and 254 differentially accumulated metabolites (DAMs) in ‘Pacific Early’ and ‘Jilv3’ under drought stress compared with normal watering, respectively. The amino acid, flavonoid, organic acid, and soluble sugar contents were more significantly enhanced in ‘Jilv3’ than in ‘Pacific Early’. According to the metabolome and transcriptome analysis, in ‘Jilv3’, the energy supply of the TCA cycle was improved, and flavonoid biosynthesis increased. As a result, its adaptability to drought stress improved.

**Conclusions:**

These findings help to better reveal the molecular mechanism underlying how asparagus responds to drought stress and improve researchers’ ability to screen drought-tolerant asparagus varieties as well as breed new varieties.

**Supplementary Information:**

The online version contains supplementary material available at 10.1186/s12870-024-05286-z.

## Introduction

Drought is a leading environmental factor affecting the growth and development of plants [1]. With global warming, water scarcity has become a major challenge for agricultural production. Stress caused by drought affects the growth of plants, causing increased reactive oxygen species (ROS), stomatal closure, decreased metabolism, and reduced photosynthesis [2–4]. To mitigate the deleterious effects of drought, plants have evolved multiple mechanisms to cope with drought, including enhancing antioxidant defense systems [5], maintaining the structure and characteristics of the biological membrane [6], and accumulating more osmoregulatory substances in cells [7, 8].

Drought stress disrupts the balance of ROS metabolism in plants and triggers oxidative stress reactions [5]. Catalase (CAT), peroxidase (POD), and superoxide dismutase (SOD) are essential in ROS scavenging [9]. In plants, nonenzymatic defense antioxidants, such as ascorbic acid and glutathione, effectively and efficiently scavenge ROS and enhance redox sensing and signaling [10]. Flavonoids play an important buffering role in maintaining the cellular redox balance, thus enabling plants to maintain optimal energy metabolism under adverse conditions [11, 12]. Studies have shown that the accumulation of flavonoids and overexpression of key genes in the flavonoid biosynthesis pathway enhance the drought resistance and antioxidant activity of wheat and *Arabidopsis* [13, 14]. In addition, flavonoid glycosylation can effectively scavenge ROS in plants. Previous research showed that large amounts of flavonoid glycosylation accumulated in a drought-tolerant genotype of Tibetan hulless barley and that the expression of glucosyltransferase genes was induced by drought stress, which further improved its drought tolerance [15].

Under drought stress, plant metabolism is disrupted, and metabolic networks are reconfigured [16, 17]. Amino acids (notably proline), betaines, organic acids, soluble proteins, and soluble sugars, all of which are osmoregulatory substances, change during this process [18, 19]. Studies have shown that the contents of allantoin, galacturonic acid, gluconic acid, and glucose are positively correlated with drought resistance in various rice genotypes [20], and the high levels of amino acids, alkaloids, and organic acids in drought-tolerant genotypes of wheat following drought treatment assist in explaining their strong drought tolerance [21]. Under drought stress, metabolic processes, such as the citrate cycle (TCA cycle), glucose metabolism, and glycolysis/gluconeogenesis, change to different degrees. These metabolisms correspond closely to drought resistance [21–23].

Asparagus (*Asparagus officinalis* L.) is an important economic crop with rich nutritional and medicinal values [24]. Asparagus has strong drought resistance, but there are significant differences in drought resistance among the different genotypes, and the same genotype exhibits varying degrees of drought resistance at various stages of growth. Current studies on asparagus drought resistance have focused on biochemistry, morphology, and physiology. Research on metabolic regulation and transcription has not been reported. In this study, metabolome profiling and transcriptome sequencing analysis were conducted on two varieties of asparagus with different levels of drought resistance. Multiple DAMs and DEGs were identified. A joint analysis was performed to determine the key pathways through which asparagus responds to drought stress. These findings prove a basis to clarify the molecular mechanism of how asparagus responds to drought stress.

## Materials and methods

### Materials and design

The experiment was conducted using an artificial climate chamber at the Institute of Economic Crops of the Hebei Academy of Agriculture and Forestry Sciences between March and August 2022. The drought-tolerant ‘Jilv3’ and drought-sensitive ‘Pacific Early’ asparagus varieties were obtained from the Institute of Economic Crops of the Hebei Academy of Agriculture and Forestry Sciences. The two varieties were evaluated in a pot experiment in 2019 (Table S1). The ‘Jilv3’ and ‘Pacific Early’ seeds were sown in plastic pots (6 cm in diameter; 6 cm in depth). The pots were filled with 3 g of compound fertilizer (15% N, 15% P_2_O_5_, and 15% K_2_O) and 100 g of air-dried cinnamon soil. The soil water content (SWC) was monitored using a soil moisture sensor inserted into the soil. All of the seedlings were watered normally after sowing to maintain the SWC at 75–80% until drought stress treatment was initiated. Starting on the 71st day after sowing, the plants were divided into two watering treatment groups: (1) the normal watering (control) group and (2) the drought stress treatment group. The SWC in the control group was maintained at 75–80% by watering every 2 days. The water supply was stopped in the drought treatment group. Leaves were collected from the top half of the tallest stems and frozen in liquid nitrogen on day 12 after treatment, when the SWC of the drought treatment group was 25–28% [25]. The leaves were stored at − 80 °C, including samples collected from the ‘Pacific Early’ control group (PCK), the ‘Pacific Early’ drought stress treatment group (PDS), the ‘Jilv3’ control group (JCK), and the ‘Jilv3’ drought stress treatment group (JDS). Each group had three replicates with nine seedlings per replicate.

### Determination of physiological and biochemical indices

The anthrone colorimetric method was employed to determine the soluble sugar content [26]. The soluble protein content was obtained using the Coomassie Brilliant Blue G250 method [27]. The malondialdehyde (MDA) content and POD, SOD, and CAT activities were calculated using an MDA assay kit, POD assay kit, SOD assay kit, and CAT assay kit, respectively. The kits were purchased from Suzhou Comin Biotechnology Co., Ltd. (Suzhou, China) and used according to the manufacturer’s instructions.

### RNA extraction and sequencing

The RNAprep Pure Plant Kit (Tiangen, Beijing, China) was employed to extract the total RNA from 12 samples. To detect the RNA quality and concentration for cDNA library preparation, an Agilent 2100 Bioanalyzer was used (Agilent Technologies, Inc., Santa Clara, CA, USA). The prepared library was sequenced on the Illumina HiSeq 2500 platform (Illumina, Inc., San Diego, CA, USA). Reads with adapters, poly-N, and low-quality reads were removed from the raw data. The resulting high-quality reads were compared with the garden asparagus reference genome (GCA_001876935.12007) using HISAT2. The BMKCloud platform (www.biocloud.net) was employed for DEG analysis. The fragments per kilobase of transcript per million fragments mapped (FPKM) was obtained using StringTie software. DEGs were identified in DESeq2 with screening criteria of |Log_2_FC| > 1 and false discovery rate (FDR) < 0.05. DEG functional analysis was performed using Gene Ontology (GO) and the Kyoto Encyclopedia of Genes and Genomes (KEGG).

### Quantitative real-time polymerase chain reaction (RT-qPCR) analysis

Nine DEGs were selected for RT-qPCR based on their putative functions involved in scavenging ROS, phenylpropanoid biosynthesis, the TCA cycle, and glycolysis/gluconeogenesis. A Fast Quant RT Kit (Tiangen, Beijing, China) was used to reverse-transcribe RNA into cDNA. Primer Premier v5.0 (Premier Biosoft, Palo Alto, CA, USA) was used for primer design, as shown in Table S2. A Bio-Rad CFX96 real-time PCR detection system (Bio-Rad, USA) with TransStart Top Green qPCR SuperMix (TransGen Biotech, Beijing, China) was used to perform RT-qPCR. The internal reference gene used was selected ubiquitin-long tail fusion (GenBank: X66875.1). The relative transcript abundance was calculated using the 2^–ΔΔCT^ method [28]. For each gene, quantitative expression analysis was performed with three biological and technical replicates.

### Metabolite extraction, detection, and analysis

Metabolite extraction, detection, and analysis were completed at Wuhan MetWare Biotechnology Co., Ltd. (Wuhan, China). The frozen samples were ground, and 100 mg of freeze-dried powder was dissolved in 1.2 mL of 70% methanol solution at 4 °C to extract samples. The supernatant was filtered with a microporous membrane (0.22 μm pore size) following centrifugation for 4 min at 10,000 rpm. UPLC-MS/MS (UPLC, SHIMADZU Nexera X2; MS, Applied Biosystems 4500Q TRAP) was used to analyze the samples [24]. Metabolite qualitative analysis was conducted using the MetWare database (http://www.metware.cn/) with secondary spectrum information. The multiple reaction monitoring mode (MRM) was employed to conduct metabolite quantification analysis. Principal component analysis (PCA) was performed to analyze sample differences. For all samples, orthogonal partial least-squares discriminant analysis (OPLS-DA) was performed, and DEMs were screened on the basis of fold change (FC; ≥2 or ≤ 0.5) and variable importance in projection (VIP; ≥1). After metabolites were identified, the KEGG database was used for annotation.

### Statistical analysis

The experiment was conducted using a completely random block design. Significance analysis was performed using one-way analysis of variance (ANOVA) and Duncan’s multiple range test in SPSS Statistics 22.0 software, where *P* < 0.05 indicated a significant difference. All values are represented as the mean of three biological replicates ± standard deviation (SD).

## Results

### Physiological responses of ‘Jilv3’ and ‘Pacific Early’ to drought stress

**Fig. 1:**
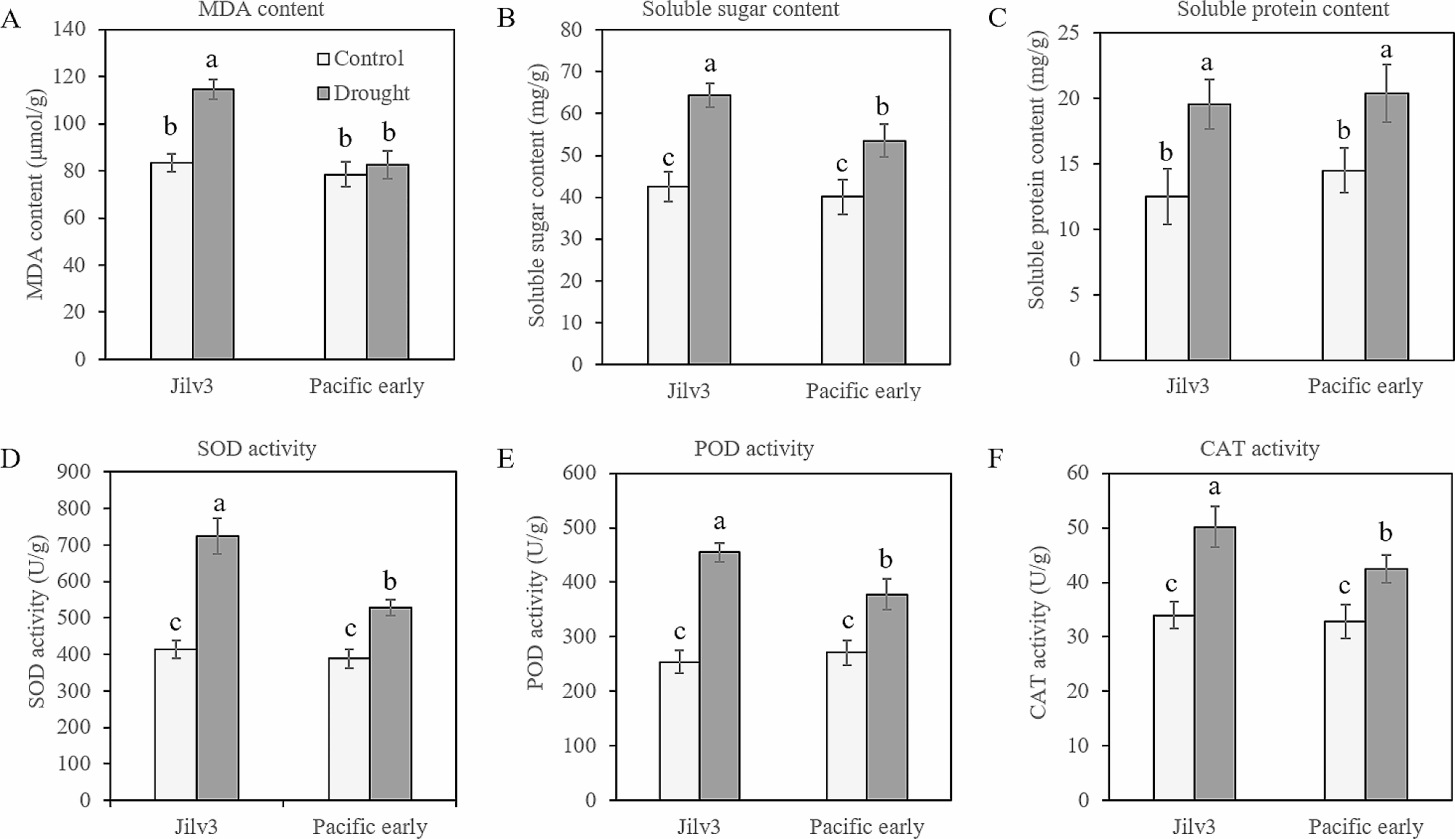
Physiological and biochemical changes of ‘Jilv3’ and ‘Pacific Early’ asparagus seedlings under drought stress. (**A**) Malondialdehyde (MDA), (**B**) soluble sugar, and (**C**) soluble protein contents. (**D**) Superoxide dismutase (SOD), (**E**) peroxidase (POD), and (**F**) catalase (CAT) enzyme activities

The physiological indicators are shown in Fig. 1. The results showed that the activities of POD, SOD, and CAT in ‘Jilv3’ increased under drought stress by 79.13%, 76.46%, and 47.65%, respectively, and in ‘Pacific Early’, these values increased by 39.48%, 35.73%, and 29.57%, respectively. The activities of POD, SOD, and CAT under drought stress increased in both asparagus varieties; however, the increases in ‘Jilv3’ (2.14-, 2.00-, and 1.61-fold respectively) were greater than those in ‘Pacific Early’. In both asparagus varieties, soluble sugar and soluble protein increased significantly under drought stress. The soluble sugar and soluble protein contents in ‘Jilv3’ increased by 51.5% and 56.8%, respectively, while in ‘Pacific Early’, the soluble sugar and protein contents increased by 33.4% and 40.7%, respectively. The soluble sugar content in ‘Jilv3’ increased considerably more than it did in ‘Pacific Early’. The MDA content increased significantly in ‘Jilv3’ (37.3%) following drought stress but not in ‘Pacific Early’.

### Transcriptome analysis of ‘Jilv3’ and ‘Pacific Early’ under drought stress

Twelve cDNA libraries were designed for RNA-seq analysis to evaluate the effect of drought stress on the asparagus transcriptome. Using the measured data, this study generated 265.02 million pairs of paired-end sequences with strict quality control to obtain 79.06 Gb of clean data. The GC percentage was between 46.64% and 48.63% and the Q30 ratio was more than 93.25%. The comparison rate between the transcriptome data and the asparagus reference genome was 83.27–85.88% (Table S3).

### DEG identification and analysis

**Fig. 2:**
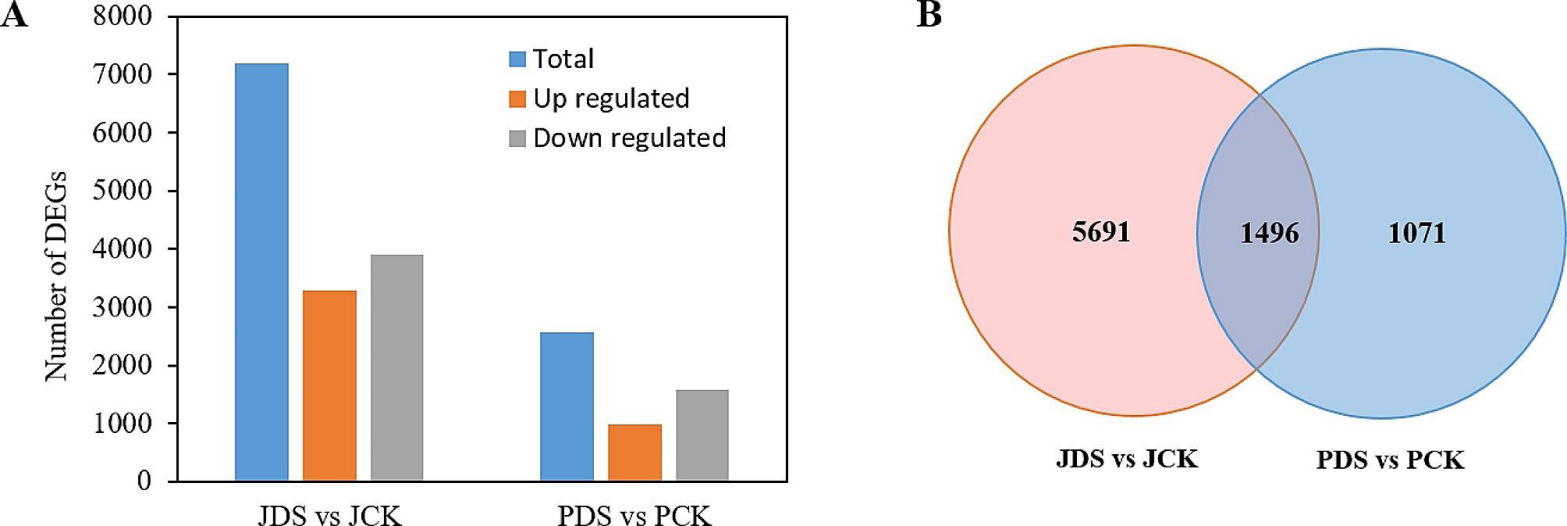
Transcriptome analysis of drought stress response in two asparagus cultivars. (**A**) DEGs in JDS versus JCK and PDS versus PCK. (**B**) Specific and common DEGs in JDS versus JCK and PDS versus PCK. JCK, ‘Jilv3’ with normal watering; JDS, ‘Jilv3’ with drought stress treatment; PCK, ‘Pacific Early’ with normal watering; and PDS, ‘Pacific Early’ with drought stress treatment

In total, 2,567 (990 upregulated and 1,577 downregulated) DEGs and 7,187 (3,280 upregulated and 3,907 downregulated) DEGs were identified in PDS versus PCK and in JDS versus JCK, respectively, after applying the criteria of FDR < 0.05 and |log_2_FC| ≥ 1 (Fig. 2A and Table S4). The number of DEGs in ‘Pacific Early’ was much lower than that in ‘Jilv3’, which showed that ‘Jilv3’ responded more strongly to drought stress. As shown in the Venn diagram in Fig. 2B, there were 5691 specific DEGs in JDS versus JCK, 1071 specific DEGs in PDS versus PCK, and 1496 DEGs common to both comparison groups. Nine genes related to key metabolic pathways were randomly selected for RT-qPCR analysis to evaluate the RNA-seq data accuracy. These nine genes followed similar trends in RT-qPCR gene expression to those in the RNA-seq data (Fig. S1). 

**Fig. 3:**
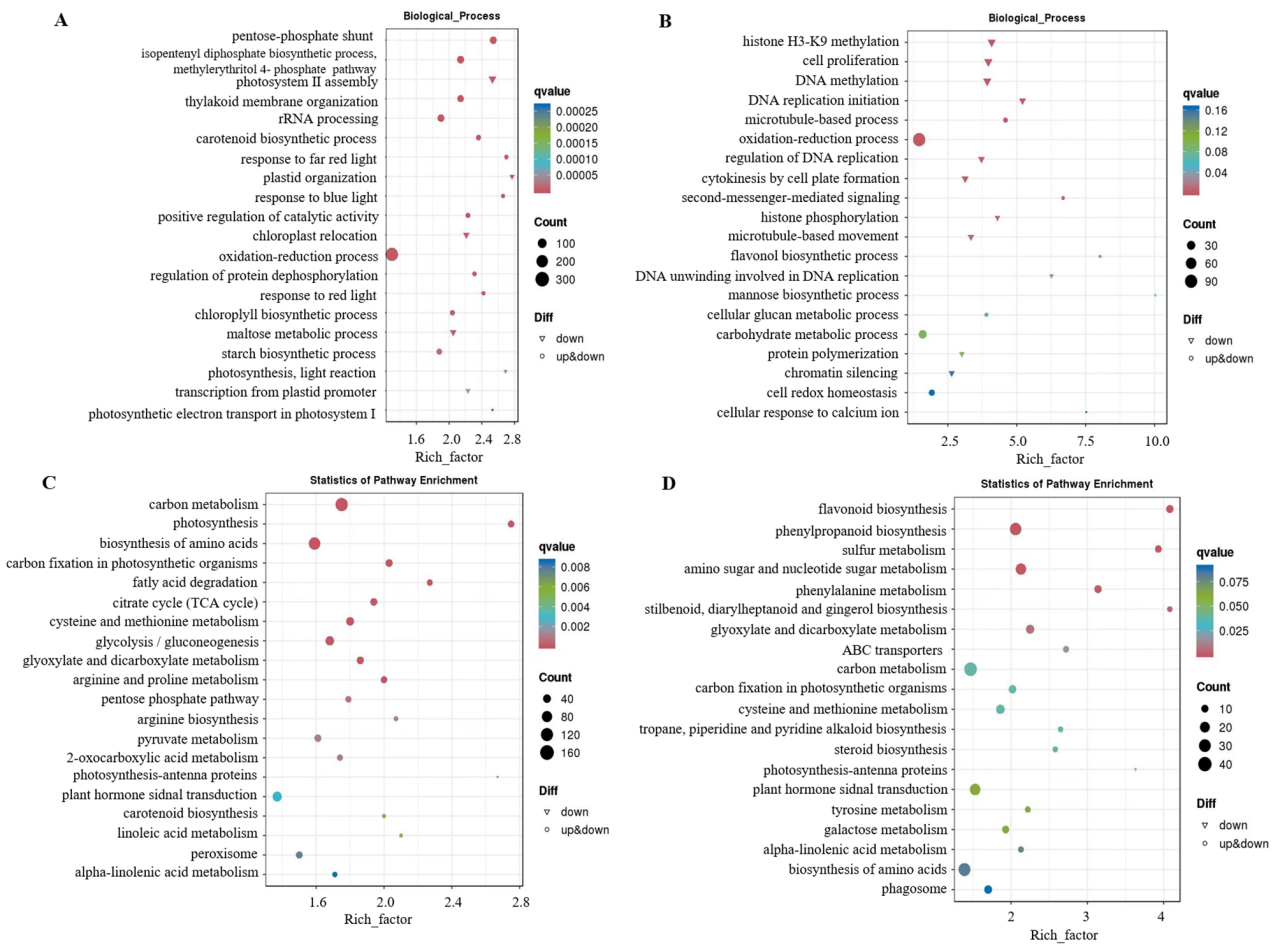
GO and KEGG enrichment analysis of DEGs. GO enrichment analysis of (**A**) JDS versus JCK and (**B**) PDS versus PCK. KEGG enrichment analysis of (**C**) JDS versus JCK and (**D**) PDS versus PCK. JCK, ‘Jilv3’ with normal watering; JDS, ‘Jilv3’ with drought stress treatment; PCK, ‘Pacific Early’ with normal watering; and PDS, ‘Pacific Early’ with drought stress treatment

GO and KEGG analyses were performed to identify the DEG functions. DEGs in JDS versus JCK were enriched in 630 GO terms. Among these, 236, 241, and 153 DEGs were related to the cellular component (CC), biological process (BP), and molecular function (MF) categories, respectively. The DEGs in PDS versus PCK were enriched in 308 GO terms, of which 20, 183, and 105 DEGs were related to CC, BP, and MF, respectively (Table S5). Many of the DEGs in the top 20 enriched terms in BP (according to the ascending order of q values) were enriched in the carbohydrate metabolism–related and oxidation–reduction processes in both groups. In addition, in ‘Jilv3’, many genes were enriched in photosynthesis-related processes, including photosystem II assembly, thylakoid membrane organization, photosynthesis, light reaction, and photosynthetic electron transport in the photosystem. Many ‘Pacific Early’ genes were enriched in DNA and histone modification-related processes, such as cell proliferation, DNA replication initiation, histone H3-K9 methylation, cytokinesis by cell plate formation, and DNA methylation (Fig. 3A, B). As to CC and MF, the top 5 enriched terms of DEGs in JDS versus JCK were also closely related to photosynthesis-related and oxidation–reduction processes, such as the terms of chloroplast thylakoid membrane, chloroplast envelope, and chloroplast stroma in CC, and the terms of oxidoreductase activity and oxidoreductase activity (acting on the CH-NH group of donors) in MF. While in PDS versus PCK, the top 5 enriched CC terms were integral component of membrane, microtubule, integral component of plasma membrane, microtubule cytoskeleton, and MCM complex, and the top 5 enriched MF terms were ubiquitin protein ligase activity, ubiquitin-protein transferase activator activity, anaphase-promoting complex binding, O-acetyltransferase activity, and sequence-specific DNA binding RNA polymerase II transcription factor activity (Table S5).

The results of KEGG enrichment analysis showed that DEGs were enriched in 112 KEGG pathways for PDS versus PCK and 122 KEGG pathways for JDS versus JCK (Table S6). The top 20 enriched pathways were arranged in ascending order of q value. Many of the DEGs were enriched in amino acid biosynthesis, carbon metabolism, plant hormone signal transduction, and phenylpropanoid biosynthesis in both comparison groups. In JDS versus JCK, many DEGs were enriched in energy metabolism–related pathways, such as the TCA cycle, glycolysis/gluconeogenesis, and pyruvate metabolism. Many DEGs in the case of PDS versus PCK were enriched in flavonoid biosynthesis and phenylpropanoid biosynthesis pathways (Fig. 3C, D).

### Metabolomics analysis of ‘Jilv3’ and ‘Pacific Early’ under drought stress

**Fig. 4:**
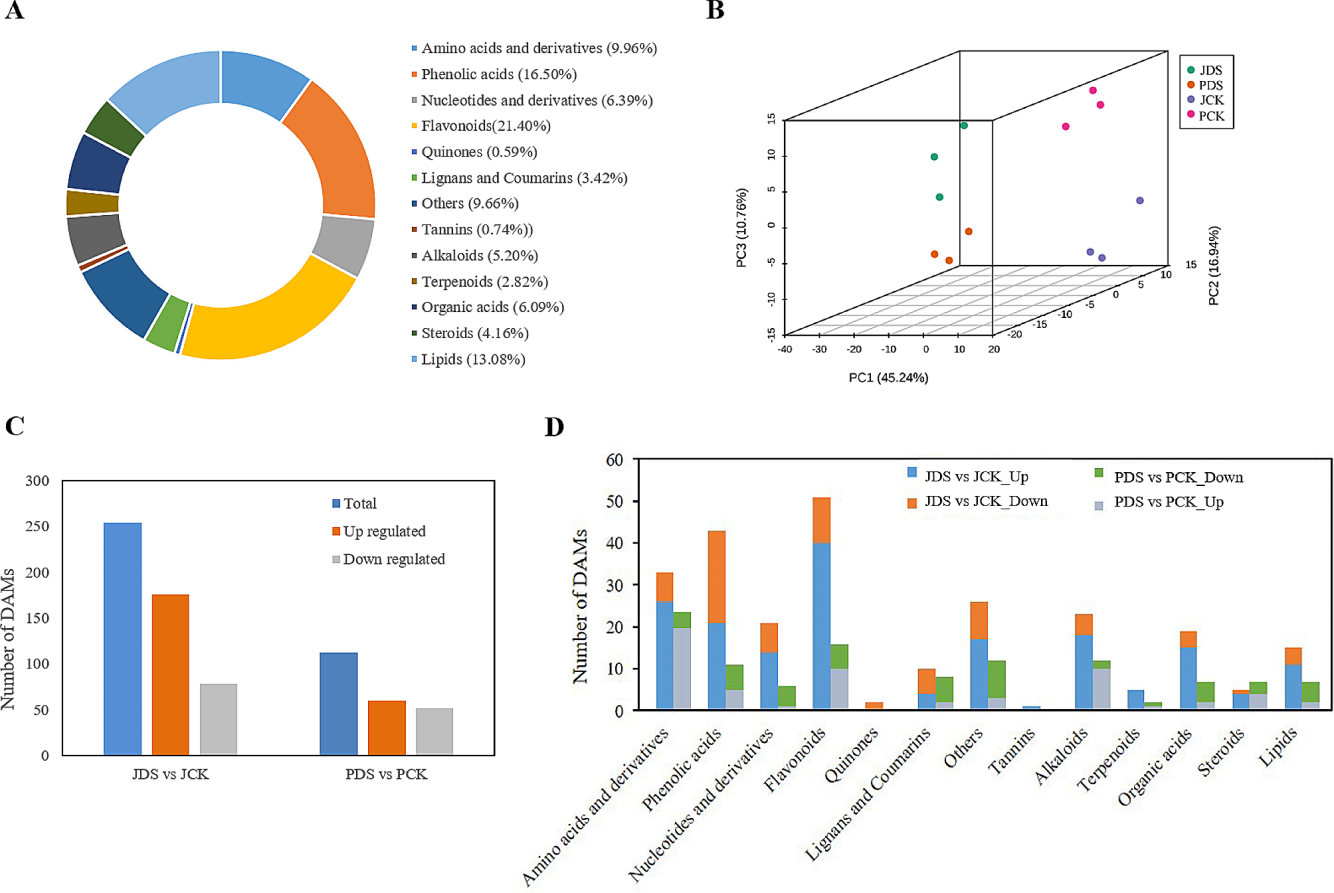
Metabolomics analysis of ‘Jilv3’ and ‘Pacific Early’ asparagus seedlings under drought stress. (**A**) Total metabolite visualization. (**B**) Metabolomic data clustering from PCA. (**C**) The DAMs in JDS versus JCK and PDS versus PCK. (**D**) DAM classification

UPLC-MS/MS was employed to conduct a targeted metabolomics analysis and metabolite accumulation under drought stress was compared in ‘Jilv3’ and ‘Pacific Early’. Among the samples, 673 metabolites were detected, comprising 111 phenolic acids, 67 amino acids and derivatives, 43 nucleotides and derivatives, 23 lignans and coumarins, 144 flavonoids, four quinones, 35 alkaloids, five tannins, 19 terpenoids, 88 lipids, 41 organic acids, 28 steroids, and 65 others (Fig. 4A and Table S7). PCA revealed a distinct separation among the samples (Fig. 4B).

A total of 112 DAMs (60 increased and 52 decreased) were detected for PDS versus PCK, while 254 DAMs (176 increased and 78 decreased) were detected for JDS versus JCK based on the criteria of VIP ≥ 1 and FC ≥ 2 or ≤ 0.5. The comparison groups shared 76 common DAMs (Fig. 4C and Table S8). Following drought occurrence, metabolite accumulation in ‘Jilv3’ and ‘Pacific Early’ showed differential responses (Fig. 4D). For example, proline increased 25.7-fold in ‘Jilv3’ under drought conditions, but it did not significantly change in ‘Pacific Early’. Although the contents of valine, leucine, isoleucine, histidine, and phenylalanine increased significantly in both varieties following drought stress, the increase was higher in ‘Jilv3’ than in ‘Pacific Early’. In ‘Jilv3’, the valine, leucine, isoleucine, histidine, and phenylalanine contents increased 5.25-fold, 5.88-fold, 5.92-fold, 5.49-fold, and 7.87-fold, respectively, while, in ‘Pacific Early’, these contents increased 2.35-fold, 2.75-fold, 2.93-fold, 2.05-fold, and 3.56-fold, respectively. After drought treatment, the contents of 10 carbohydrates, including glucose, fructose, trehalose, and melibiose, increased significantly in ‘Jilv3’ with an increase range of 2.03-fold to 25.04-fold. For example, the mannitol, glucose, fructose, trehalose, and melibiose contents increased 25.04-fold, 2.79-fold, 2.68-fold, 2.86-fold, and 5.51-fold, respectively. In contrast, only the sorbitol content increased significantly in ‘Pacific Early’ (2.20-fold). Under drought stress, the contents of 15 of 19 differentially accumulated organic acids increased significantly in ‘Jilv3’ (by 2.08-fold to 16.07-fold), including citrate (4.17-fold), isocitrate (2.08-fold), malate (2.40-fold), and succinate (2.38-fold), while only the contents of aminocaproic acid and diethyl phosphate significantly increased in ‘Pacific Early’ (4.31- and 2.06-fold, respectively). After drought treatment, the contents of 40 flavonoids increased significantly in ‘Jilv3’ (by 2.00-fold to 5.61-fold), while only 10 flavonoids significantly increased in ‘Pacific Early’ (by 2.05-fold to 4.63-fold) (Table S8).

**Fig. 5:**
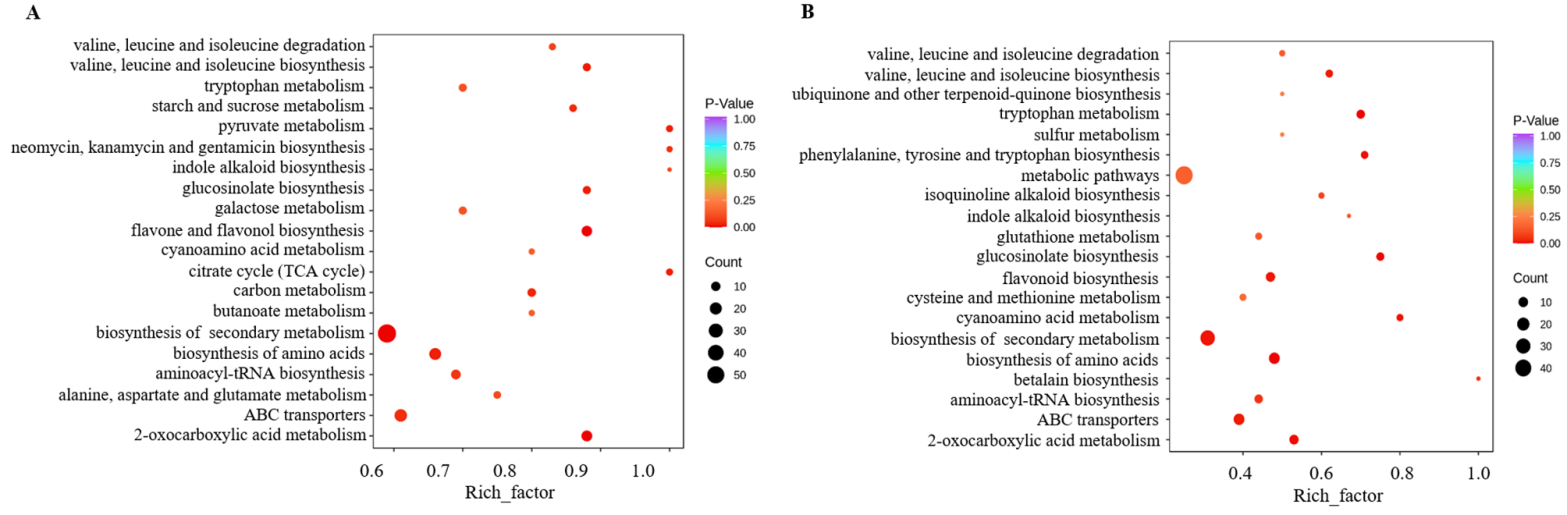
KEGG and GO metabolism pathway categories of DAMs. (**A**) JDS versus JCK. (**B**) PDS versus PCK. JCK, ‘Jilv3’ with normal watering; JDS, ‘Jilv3’ with drought stress treatment; PCK, ‘Pacific Early’ with normal watering; and PDS, ‘Pacific Early’ with drought stress treatment

Additional KEGG analysis showed that in the case of JDS versus JCK, DAMs were enriched in 82 KEGG pathways, while in the case of PDS versus PCK, DAMs were enriched in 56 KEGG pathways (Table S9). Among the top 20 KEGG pathways, both comparison groups were enriched in ABC transporters, biosynthesis of amino acids, 2-oxocarboxylic acid metabolism, and pyruvate metabolism. Additionally, in the case of JDS versus JCK, DAMs were enriched in carbon metabolism, the TCA cycle, and flavone and flavonol biosynthesis, while for PDS versus PCK, DAMs were enriched in glutathione metabolism (Fig. 5A, B).

### Analysis of flavonoid biosynthesis pathway–related genes and metabolites

**Fig. 6:**
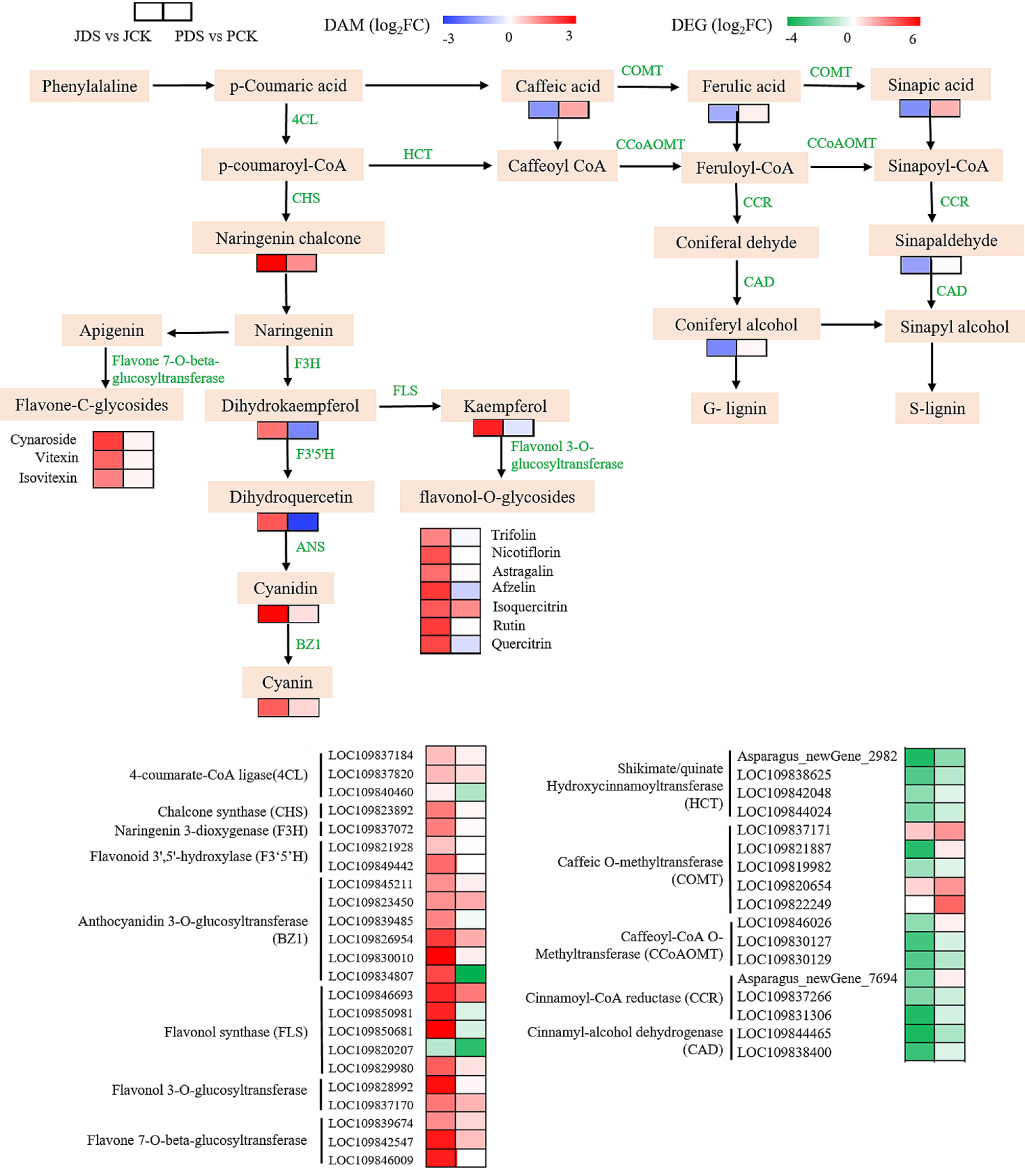
Schematic network of the flavonoid biosynthesis pathway and lignin synthesis pathway in asparagus under drought stress. Colored rectangle blocks denote the DEGs or DAMs for JDS versus JCK (left) and PDS versus PCK (right). Different colors indicate the logarithm of fold changes (Log_2_FC) of the DEGs and DAMs. JCK, ‘Jilv3’ with normal watering; JDS, ‘Jilv3’ with drought stress treatment; PCK, ‘Pacific Early’ with normal watering; and PDS, ‘Pacific Early’ with drought stress treatment

An integrated analysis of metabolomic and transcriptomic data was conducted in this study, and it was found that the lignin synthesis and flavonoid biosynthesis pathways were essential in the response of asparagus to drought stress (Fig. 6 and Table S10).

In ‘Jilv3’ under drought stress, 22 key genes at the transcriptional level were involved in the flavonoid biosynthesis pathway and were upregulated. They were as follows: one chalcone synthase (*CHS*) gene, one naringenin 3-dioxygenase (*F3H*) gene, one flavonoid 3’,5’-hydroxylase (*F3’5’H*) gene, two anthocyanidin synthase (*ANS*) genes, two 4-coumarate-CoA ligase (*4CL*) genes, two flavonol 3-O-glucosyltransferase genes, three flavone 7-O-beta-glucosyltransferase genes, four flavonol synthase (*FLS*) genes, and six anthocyanidin 3-O-glucosyltransferase (*BZ*1) genes. In ‘Pacific Early’ under drought stress, only the following genes were upregulated: one *FLS* gene, one flavone 7-O-beta-glucosyltransferase gene, one flavonol 3-O-glucosyltransferase gene, and two *BZ1* genes.

By contrast, in ‘Jilv3’ under drought stress, the following 14 key genes involved in the lignin synthesis pathway were downregulated: two caffeic O-methyltransferase (*COMT*) genes, two cinnamyl-alcohol dehydrogenase (*CAD*) genes, three caffeoyl-CoA O-methyltransferase (*CCoAOMT*) genes, three cinnamoyl-CoA reductase (*CCR*) genes, and four shikimate/quinate hydroxycinnamoyltransferase (*HCT*) genes. However, only four related genes were downregulated in ‘Pacific Early’, and the downregulation range was smaller than that of ‘Jilv3’.

The metabolomics analysis of ‘Jilv3’ under drought stress showed that multiple metabolites in the flavonoid biosynthesis pathway were significantly increased, including anthocyanin, dihydrokaempferol, dihydroquercetin, flavone-C-glycosides, flavone/flavonol-O-glycosides, kaempferol, and naringenin chalcone. In ‘Pacific Early’, the only significant increases were found in naringenin chalcone and one flavonol-O-glycoside (isoquercitrin).

In ‘Jilv3’ under drought stress, the lignin pathways exhibited significantly decreased contents of hydroxycinnamic acid (caffeic acid, ferulic acid, and sinapic acid), monolignols (coniferyl alcohol), and monolignol precursors (sinapinaldehyde). However, no significant changes, were detected in ‘Pacific Early’. The integrated analysis of metabolome and transcriptome suggests that enhanced drought tolerance in ‘Jilv3’ may be a result of enhanced flavonoid biosynthesis and the reprogramming of the metabolic flow from lignin synthesis to flavonoid biosynthesis.

### Analysis of TCA cycle–related genes and metabolites

**Fig. 7:**
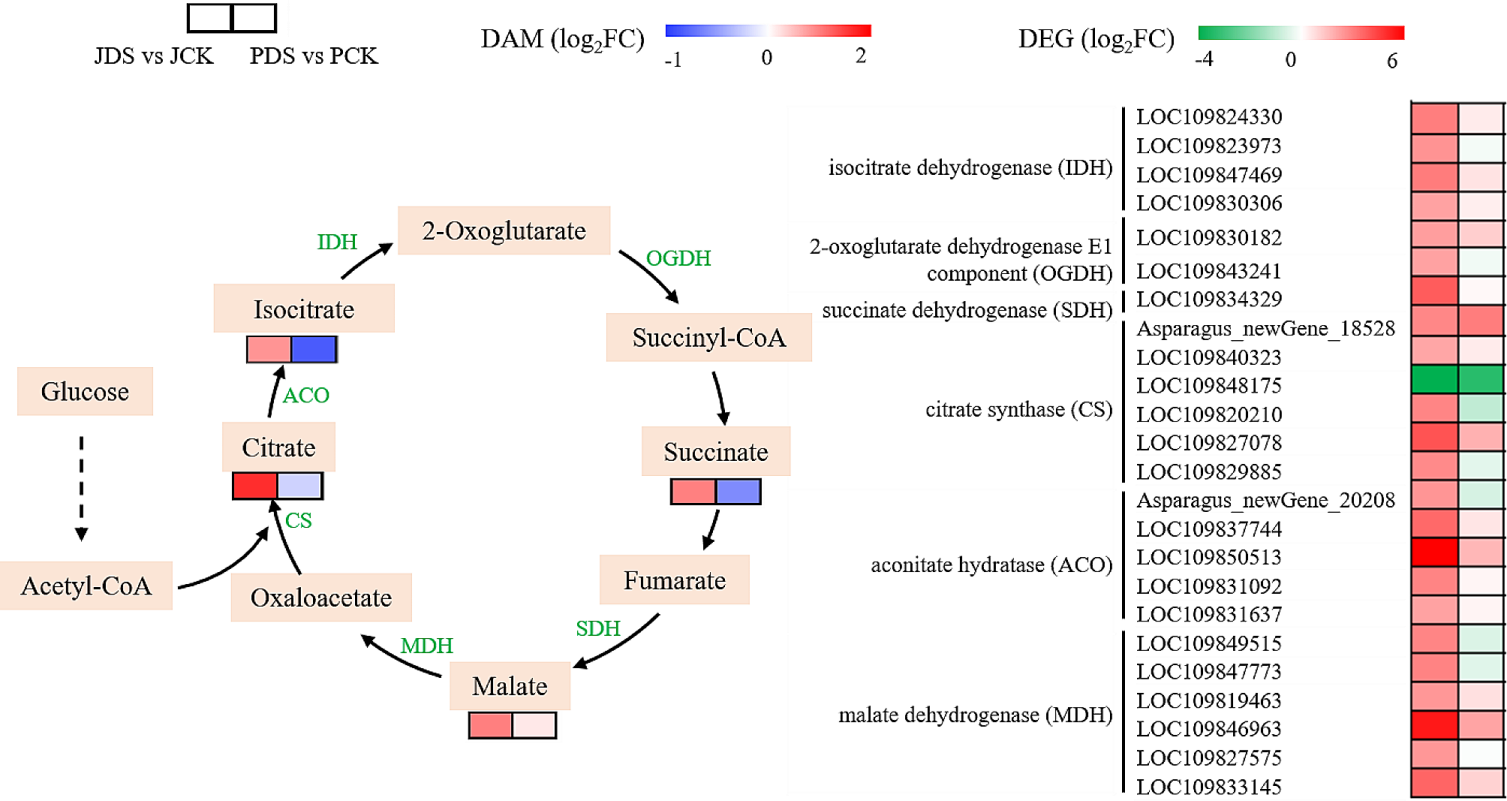
The TCA cycle in asparagus under drought stress. The two columns of rectangular blocks represent the DEGs or DAMs of JDS versus JCK (left) and PDS versus PCK (right). Different colors indicate the logarithm of fold changes (Log_2_FC) of the DEGs and DAMs. JCK, ‘Jilv3’ with normal watering; and JDS, ‘Jilv3’ with drought stress treatment; PCK, ‘Pacific Early’ with normal watering; PDS, ‘Pacific Early’ with drought stress treatment

Multiple genes involved in the TCA cycle were closely related to drought stress at the transcription level. Differential response patterns of TCA cycle-related genes were observed in ‘Jilv3’ and ‘Pacific Early’ under drought stress (Fig. 7 and Table S10). For example, in ‘Jilv3’, the following genes were upregulated: one succinate dehydrogenase (*SDH*) gene, two 2-oxoglutarate dehydrogenase E1 component (*OGDH*) genes, four isocitrate dehydrogenase (*IDH*) genes, five aconitate hydratase (*ACO*) genes, five citrate synthase (*CS*) genes, and six malate dehydrogenase (*MDH*) genes. In ‘Pacific Early’, only the following were upregulated: one *MDH* gene and two *CS* genes. According to the metabolomic data, in ‘Jilv3’ under drought stress, the levels of TCA cycle intermediates, including citrate, isocitrate, malate, and succinate, were significantly increased. In ‘Pacific Early’, these levels did not change significantly, which suggests that the TCA cycle is essential in protecting asparagus from the effects of drought stress.

## Discussion

Plant growth is affected by drought stress, and plants adapt to drought environments by activating complex regulatory mechanisms [29, 30]. We conducted a joint analysis of the metabolome and transcriptome to evaluate DAMs and DEGs in two varieties of asparagus with different levels of tolerance to drought stress. The molecular mechanisms of the effect of drought stress on asparagus were identified.

Under drought stress, the ROS content in plants increases significantly, leading to oxidative damage to cells [5]. POD, SOD, and CAT are essential in the maintenance of cellular ROS homeostasis [9]. The results of the present study showed that POD, SOD, and CAT activities in both asparagus varieties under drought stress increased significantly. The increase in ‘Pacific Early’, however, was significantly lower than that in ‘Jilv3’. In ‘Jilv3’, multiple genes encoding POD, SOD, and CAT were upregulated (Table S11). This suggested that the drought-tolerant genotype was able to rapidly activate the ROS clearance system after drought stress, which may contribute to the high enzyme activity of SOD, POD, and CAT, thus leading to a stronger ROS removal ability [31, 32]. According to the transcriptome data, the positive role of these activities alleviated drought-related cellular oxidative damage.

Flavonoids are the main secondary metabolites of the phenylpropane metabolic pathway and are widely involved in abiotic stress in plants. Drought tolerance in some plants is enhanced by the accumulation of excessive flavonoids [13]. In this study, the content of anthocyanins and kaempferol in ‘Jilv3’ increased significantly following drought treatment. Additionally, the expression of several key genes (*CHS, F3H, FLS*, and *ANS*) in the flavonoid synthesis pathway was upregulated, which was consistent with the reports of other species, including maize and Arabidopsis [13, 14]. Therefore, it is speculated that the upregulated expression of *CHS* and *4CL* genes leads to the increased accumulation of naringenin chalcone and kaempferol and their conversion into flavonoid glycoside and anthocyanin in drought-tolerant varieties [33, 34]. Flavonoid glycosides, including apigenin glucoside derivatives, kaempferol glucoside derivatives, and quercetin glucoside derivatives, play an important role in clearing ROS and alleviating oxidative stress under abiotic stress [35]. After drought treatment, the contents of flavone-C-glycosides and kaempferol in wheat were increased, and related genes were also upregulated [34]. In *Arabidopsis thaliana*, drought significantly increased the accumulation of naringenin chalcone and isoquercitrin [13]. The drought tolerance decreased after the elimination of the flavonoid glycoside biosynthesis associated transcription factor MYB12 in *A*. *thaliana* [36]. Glucosyltransferase is a key gene in flavonoid glycoside biosynthesis [15]. In ‘Jilv3’ under drought stress, the expression levels of flavone 7-O-beta-glucosyltransferase and flavonol 3-O-glucosyltransferase genes were upregulated, which promotes flavonoid glycoside biosynthesis. The accumulation of flavone-C-glycosides and flavone/flavonol-O-glycosides was also observed. These results suggest that ‘Jilv3’ may increase the accumulation of flavonoid glycosides by upregulating the expression of glucosyltransferase genes in response to drought stress. Therefore, flavonoid accumulation in ‘Jilv3’ may contribute to its enhanced drought resistance.

Several metabolic pathways commonly start at phenylpropane metabolism, including flavonoid and lignin metabolism. This pathway generates precursors for downstream metabolic branches. A competitive relationship exists between every branch for the substrates. Under abiotic stress, the downstream metabolic branches of phenylpropanoid metabolism undergo reprogramming [37, 38]. In hulless barley (*Hordeum vulgare* L.), phenylpropanoid metabolism was regulated under drought stress from lignin synthesis reprogramming to flavonoid biosynthesis [15]. In this study, genes related to lignin synthesis and flavonoid biosynthesis were found to be significantly downregulated and upregulated under drought stress, respectively, especially in ‘Jilv3’. The accumulation of corresponding metabolites related to lignin synthesis and flavonoid biosynthesis also decreased and increased, respectively. These results confirmed the regulation of metabolic flow from lignin synthesis to flavonoid biosynthesis in the drought-tolerant genotype in response to drought stress. Although the results of the present study contradict a previous finding that lignin was involved in abiotic stress resistance in crops, including maize and watermelon [39, 40], the present results are consistent with reports on white birch, *Arabidopsis*, and Tibetan hulless barley, which showed a negative correlation between lignin levels and drought resistance [15, 41, 42]. Therefore, the changing direction of metabolic flow was different among plants in adaption to drought stress, while for asparagus, the flow toward flavonoid biosynthesis may be advantageous in response to drought stress.

Starch and sucrose metabolism play an important role in plant drought resistance physiology, and the accumulation of soluble sugars and sugar alcohols in sugar metabolism can enhance the osmoprotective ability of cells [43]. Glucose and fructose are important energy sources for plant growth under drought stress [44]. In ‘Jilv3’, the glucose and fructose content increased with the enhancement of sucrose hydrolysis under drought stress. Similarly, mannitol, sorbitol, and melibiose also accumulated significantly in ‘Jilv3’, which is consistent with the changes in gene expression related to sugar metabolism. Trehalose acts as an osmolarity, cell membrane, and protein protectant and can improve plant drought resistance [45, 46]. Under drought stress, the expression of *TPS* and *TPP* genes in ‘Jilv3’ was upregulated, and the content of trehalose increased significantly. Therefore, the regulation of DEGs and the accumulation of metabolites in sugar metabolism help ‘Jilv3’ maintain higher cellular homeostasis [43]. In contrast, genes and metabolites related to sugar metabolism were less changed in ‘Pacific Early’, indicating that ‘Pacific Early’ failed to produce more osmoregulatory substances to resist drought stress. The TCA cycle is an important component of plant energy metabolism [21, 23, 47]. In this study, in ‘Jilv3’ under drought stress, the TCA cycle was enhanced by the upregulation of related DEGs, including *SDH*, *CS*, *SDH*, and *IDH*, which could efficiently improve the utilization of malic acid, citric acid, and succinic acid. We also observed a significant increase in related metabolites (citrate, isocitrate, succinate, and malate), which can improve the ROS detoxification capacity, osmotic adjustment, membrane stability, and thus drought tolerance. Thus, it was found that ‘Jilv3’ under drought stress sufficiently maintained its energy supply by regulating the TCA cycle. This result is consistent with previous reports of enhanced TCA cycle under drought stress in wheat [21] and *Haloxylon* [22]. Citrate is essential in drought stress [48]. In a drought environment, excess citric acid may rapidly restart the TCA cycle and energy metabolism without the input of glycolysis products [49]. Glutamine metabolism, which involves malic acid and citric acid, is also essential for plant cell proliferation and survival under stress [50]. Collectively, ‘Jilv3’ enhances the TCA cycle to meet the energy requirements and metabolic demands during drought stress.

## Conclusion

In the present study, transcriptomic and metabolomic analyses were conducted on two varieties of asparagus (‘Jilv3’ and ‘Pacific Early’) under drought stress. In total, 2,567 and 7,187 DEGs were identified in ‘Pacific Early’ and ‘Jilv3’, respectively, while 112 and 254 DAMs were identified in ‘Pacific Early’ and ‘Jilv3’, respectively. There were more DEGs and DAMs in ‘Jilv3’ than in ‘Pacific Early’ under drought stress. Through gene expression and metabolite accumulation pattern analysis, functional annotation, and enrichment analysis, and with reference to the related literature, several essential genes, metabolites, and further possible mechanisms of asparagus involved in adaptation to drought stress were identified in this study. In summary, compared with ‘Pacific Early’, the enhanced antioxidant enzyme systems, transformed metabolic flow from lignin synthesis to flavonoid biosynthesis, and improved energy supply of ‘Jilv3’ may contribute to its strong drought tolerance. Collectively, the results of this study not only improve the understanding of the molecular mechanism of asparagus in response to drought stress, but also contribute to the screening of drought-tolerant asparagus varieties and the breeding of new varieties in the future. Moreover, the results provide potential candidate genes for further asparagus drought resistance studies.

### Electronic supplementary material

Below is the link to the electronic supplementary material.

Supplementary Material 1

Supplementary Material 2

Supplementary Material 3

Supplementary Material 4

Supplementary Material 5

Supplementary Material 6

Supplementary Material 7

Supplementary Material 8

Supplementary Material 9

Supplementary Material 10

Supplementary Material 11

Supplementary Material 12

## Data Availability

We deposited sequencing data in the Sequence Read Archive (SRA), National Center for Biotechnology Information (NCBI; accession no. PRJNA873275).

## References

[1] Yang A, Akhtar S, Li L, Fu Q (2020). Biochar mitigates combined effects of drought and salinity stress in quinoa. Agronomy.

[2] Ma X, Xia H, Liu Y (2016). Transcriptomic and metabolomic studies disclose key metabolism pathways contributing to well-maintained photosynthesis under the drought and the consequent drought-tolerance in rice. Front Plant Sci.

[3] Kiani SP, Grieu P, Maury P (2007). Genetic variability for physiological traits under drought conditions and differential expression of water stress-associated genes in sunflower (Helianthus annuus l). Theor Appl Genet.

[4] Hura T, Hura K, Ostrowska A (2002). Drought-stress induced physiological and molecular changes in plants. Int J Mol Sci.

[5] Laxa M, Liebthal M, Telman W (2019). The role of the plant antioxidant system in drought tolerance. Antioxidants.

[6] Chaves MM, Maroco JP, Pereira JS (2003). Understanding plant responses to drought-from genes to the whole plant. Funct Plant Biol.

[7] Claeys H, Inze D (2013). The agony of choice: how plants balance growth and survival under water-limiting conditions. Plant Physiol.

[8] Wang X, Wang M, Yan G (2023). Comparative analysis of drought stress-induced physiological and transcriptional changes of two black sesame cultivars during anthesis. Front Plant Sci.

[9] Su P, Sui C, Niu Y (2023). Comparative transcriptomic analysis and functional characterization reveals that the class III peroxidase gene TaPRX-2A regulates drought stress tolerance in transgenic wheat. Front Plant Sci.

[10] Mittler R (2017). ROS are good. Trends Plant Sci.

[11] Di Ferdinando M, Brunetti C, Fini A, Ahmad P (2012). Flavonoids as antioxidants in plants under abiotic stresses. Abiotic stress responses in plants.

[12] Ma X, Xu Z, Lang D (2022). Comprehensive physiological, transcriptomic, and metabolomic analyses reveal the synergistic mechanism of Bacillus pumilus G5 combined with silicon alleviate oxidative stress in drought-stressed Glycyrrhiza uralensis Fisch. Front Plant Sci.

[13] Nakabayashi R, Yonekura-Sakakibara K, Urano K (2014). Enhancement of oxidative and drought tolerance in arabidopsis by overaccumulation of antioxidant flavonoids. Plant J.

[14] Lv L, Chen X, Li H (2022). Different adaptive patterns of wheat with different drought tolerance under drought stresses and rehydration revealed by integrated metabolomic and transcriptomic analysis. Front Plant Sci.

[15] Xu C, Wei L, Huang S (2021). Drought resistance in qingke involves a reprogramming of the phenylpropanoid pathway and UDP-glucosyltransferase regulation of abiotic stress tolerance targeting flavonoid biosynthesis. J Agric Food Chem.

[16] Krasensky J, Jonak C (2012). Drought, salt, and temperature stress-induced metabolic rearrangements and regulatory networks. J Exp Bot.

[17] Wang B, Lv X (2018). Whole-transcriptome sequence analysis of Verbena bonariensis in response to drought stress. Int J Mol Sci.

[18] Paudel G, Bilova T, Schmidt R (2016). Osmotic stress is accompanied by protein glycation in Arabidopsis thaliana. J Exp Bot.

[19] Tschaplinski TJ, Abraham PE, Jawdy SS (2019). The nature of the progression of drought stress drives differential metabolomic responses in Populus deltoides. Ann Bot.

[20] Ghorbanzadeh Z, Hamid R, Jacob F (2023). Comparative metabolomics of root-tips reveals distinct metabolic pathways conferring drought tolerance in contrasting genotypes of rice. BMC Genomics.

[21] Guo R, Shi L, Jiao Y (2018). Metabolic responses to drought stress in the tissues of drought-tolerant and drought-sensitive wheat genotype seedlings. AoB Plants.

[22] Yang F, Lv G (2022). Combined analysis of transcriptome and metabolome reveals the molecular mechanism and candidate genes of Haloxylon drought tolerance. Front Plant Sci.

[23] Hu Z, He Z, Li Y (2023). Transcriptomic and metabolic regulatory network characterization of drought responses in tobacco. Front Plant Sci.

[24] Zhang X, Han C, Liang Y (2022). Combined full-length transcriptomic and metabolomic analysis reveals the regulatory mechanisms of adaptation to salt stress in asparagus. Front Plant Sci.

[25] Namaki A, Ghahremani Z, Aelaei M (2022). Morpho-physiological responses of asparagus accessions to drought stress under greenhouse condition. Gesunde Pflanzen.

[26] Jan B, Roel M (1993). An improved colorimetric method to quantify sugar content of plant tissue. J Exp Bot.

[27] Pierce J, Suelter CH (1977). An evaluation of the Coomassie brilliant blue G-250 dye-binding method for quantitative protein determination. Anal Biochem.

[28] Livak KJ, Schmittgen TD (2001). Analysis of relative gene expression data using real-time quantitative PCR and the 2–∆∆CT method. Methods.

[29] Golldack D, Li C, Mohan H (2014). Tolerance to drought and salt stress in plants: unraveling the signaling networks. Front Plant Sci.

[30] Zhao X, Huang L, Sun X (2022). Transcriptomic and metabolomic analyses reveal key metabolites, pathways and candidate genes in sophora davidii (Franch.) Skeels seedlings under drought stress. Front Plant Sci.

[31] Zhang Y, Li D, Zhou R (2019). Transcriptome and metabolome analyses of two contrasting sesame genotypes reveal the crucial biological pathways involved in rapid adaptive response to salt stress. BMC Plant Biol.

[32] Miller G, Suzuki N, Ciftci-Yilmaz S (2010). Reactive oxygen species homeostasis and signalling during drought and salinity stresses. Plant Cell Environ.

[33] Nakabayashi R, Mori T, Saito K (2014). Alternation of flavonoid accumulation under drought stress in Arabidopsis thaliana. Plant Signal Behav.

[34] Ma D, Sun D, Wang C (2014). Expression of flavonoid biosynthesis genes and accumulation of flavonoid in wheat leaves in response to drought stress. Plant Physiol Biochem.

[35] Dong N, Sun Y, Guo T (2020). UDPglucosyltransferase regulates grain size and abiotic stress tolerance associated with metabolic flux redirection in rice. Nat Commun.

[36] Wang F, Kong W, Wong G (2016). AtMYB12 regulates flavonoids accumulation and abiotic stress tolerance in transgenic Arabidopsis thaliana. Mol Genet Genomicss.

[37] Dong N, Lin H (2021). Contribution of phenylpropanoid metabolism to plant development and plant-environment interactions. J Integr Plant Biol.

[38] Ranjan A, Westrick NM, Jain S (2019). Resistance against Sclerotinia sclerotioru5m in soybean involves a reprogramming of the phenylpropanoid pathway and upregulation of antifungal activity targeting ergosterol biosynthesis. Plant Biotechnol J.

[39] Fan L, Linker R, Gepstein S (2006). Progressive inhibition by water deficit of cell wall extensibility and growth along the elongation zone of maize roots is related to increased lignin metabolism and progressive stelar accumulation of wall phenolics. Plant Physiol.

[40] Yoshimura K, Masuda A, Kuwano M (2008). Programmed proteome response for drought avoidance/tolerance in the root of a C (3) xerophyte (wild watermelon) under water deficits. Plant Cell Physiol.

[41] Lee BR, Kim KY, Jung WJ (2007). Peroxidases and lignification in relation to the intensity of water-deficit stress in white clover (Trifolium repens L). J Exp Bot.

[42] Yan J, Aznar A, Chalvin C (2018). Increased drought tolerance in plants engineered for low lignin and low xylan content. Biotechnol Biofuels.

[43] Min X, Lin X, Ndayambaza B (2020). Coordinated mechanisms of leaves and roots in response to drought stress underlying full-length transcriptome profiling in Vicia sativa L. BMC Plant Biol.

[44] Cui G, Zhang Y, Zhang W (2019). Response of carbon and nitrogen metabolism and secondary metabolites to drought stress and salt stress in plants. J Plant Biol.

[45] Lin Q, Wang S, Dao Y (2020). Arabidopsis thaliana trehalose-6-phosphate phosphatase gene TPPI enhances drought tolerance by regulating stomatal apertures. J Exp Bot.

[46] Kosar F, Akram NA, Sadiq M (2019). Trehalose: a key organic osmolyte effectively involved in plant abiotic stress tolerance. J Plant Growth Regul.

[47] Lu S, Chen Y, Wang S (2023). Combined metabolomic and transcriptomic analysis reveals key components of OsCIPK17 overexpression improves drought tolerance in rice. Front Plant Sci.

[48] Ryan EM, Duryee MJ, Hollins A (2019). Antioxidant properties of citric acid interfere with the uricase-based measurement of circulating uric acid. J Pharm BioMed Anal.

[49] Martínez-Reyes I, Chandel NS (2020). Mitochondrial TCA cycle metabolites control physiology and disease. Nat Commun.

[50] Le A, Lane AN, Hamaker M (2012). Glucose-independent glutamine metabolism via TCA cycling for proliferation and survival in B cells. Cell Metab.

