# Supplementary material for: Integrated analysis of transcriptomics and metabolomics of garden asparagus (Asparagus officinalis L.) under drought stress
Source: BMC Plant Biol. 2024 Jun 15;24:563. doi: 10.1186/s12870-024-05286-z (PMC11179350; doi:10.1186/s12870-024-05286-z)
Supplement: Supplementary file 1 — Supplementary Material 1 [file 12870_2024_5286_MOESM1_ESM.docx]

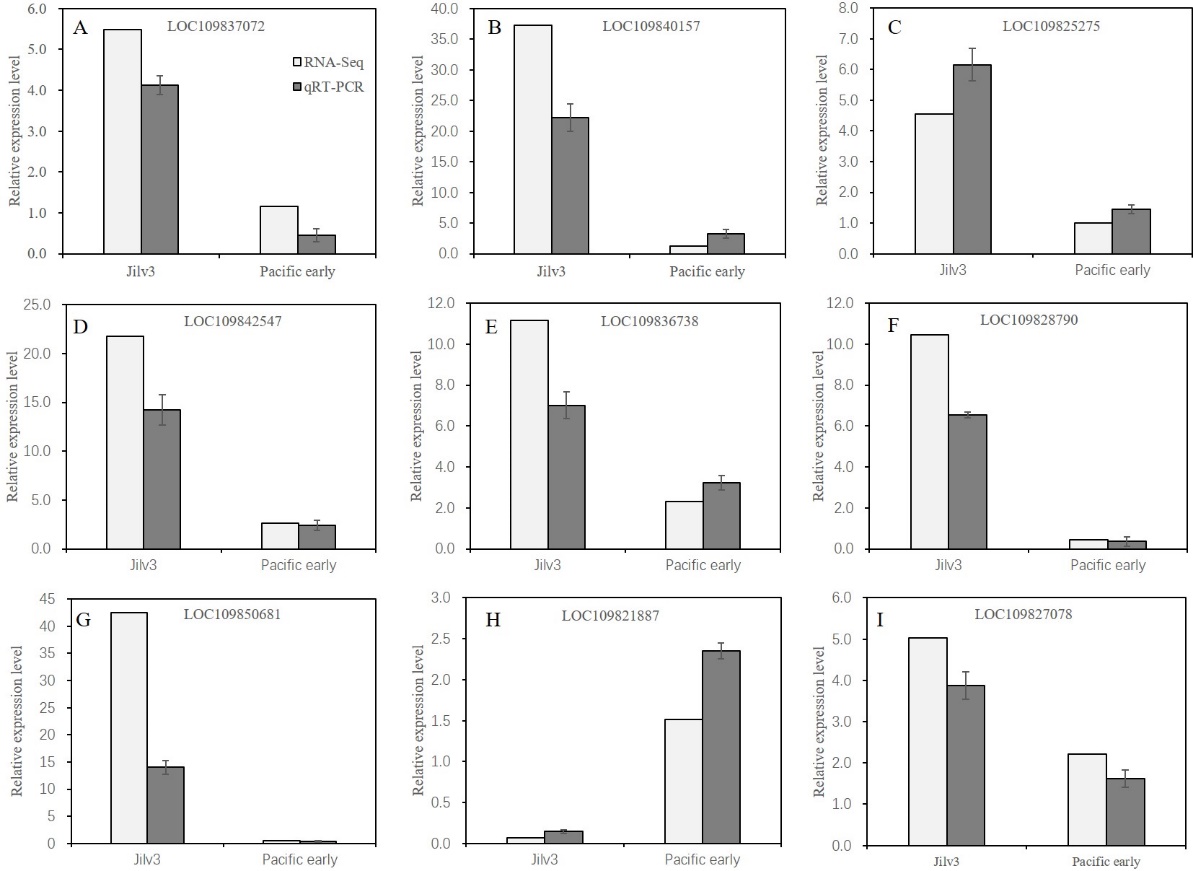


**Fig. S1.** RT-qPCR validation of 9 DEGs. The relative expression level of each gene was expressed as the fold change relative to the control (normal watering). (A)*.* *flavonoid 3-monooxygenase.* (B) *Sucrose synthase.* (C) *pyruvate kinase.* (D) *UDP-glucosyl transferase.* (E) *hexokinase.* (F) *peroxidase.* (G) *flavonol synthase.* (H) *caffeic acid 3-O-methyltransferase.* (I) *citrate synthase.*
