# Supplementary material for: Integrated analysis of transcriptomics and metabolomics of garden asparagus (Asparagus officinalis L.) under drought stress
Source: BMC Plant Biol. 2024 Jun 15;24:563. doi: 10.1186/s12870-024-05286-z (PMC11179350; doi:10.1186/s12870-024-05286-z)
Supplement: Supplementary file 2 — Supplementary Material 2 [file 12870_2024_5286_MOESM2_ESM.docx]

**Table S1. Pre-evaluation of drought-resistance in 2019**

| Materials | Plant height (cm) | | | Dry weight of plant (g) | | | Fresh weight of plant (g) | | | Drought-injury index (%) |
| --- | --- | --- | --- | --- | --- | --- | --- | --- | --- | --- |
|  | Well-watered | Drought-stressed | Decline (%) | Well-watered | Drought-stressed | Decline (%) | Well-watered | Drought-stressed | Decline (%) |  |
| Apllo | 96 | 71 | 26.04 | 180 | 150 | 16.67 | 1587 | 1208 | 23.88 | 50 |
| UC115 | 114 | 87 | 23.68 | 247 | 204 | 17.41 | 1687 | 1324 | 21.52 | 36.1 |
| UC157 | 124 | 96 | 22.58 | 269 | 221 | 17.84 | 1960 | 1578 | 19.49 | 38.9 |
| UC800 | 87 | 62 | 28.74 | 189 | 141 | 25.40 | 1266 | 854 | 32.54 | 52.8 |
| NJ1156 | 76 | 56 | 26.32 | 207 | 158 | 23.67 | 1740 | 1352 | 22.30 | 36.1 |
| Grande | 111 | 91 | 18.02 | 268 | 226 | 15.67 | 1954 | 1685 | 13.77 | 22.2 |
| Jersey night | 102 | 82 | 19.61 | 216 | 168 | 22.22 | 1574 | 1301 | 17.34 | 38.9 |
| Mondeo | 118 | 86 | 27.12 | 257 | 185 | 28.02 | 1846 | 1325 | 28.22 | 50 |
| Pacific challenger | 105 | 75 | 28.57 | 222 | 168 | 24.32 | 1746 | 1341 | 23.20 | 47.2 |
| Atlas | 98 | 82 | 16.33 | 198 | 170 | 14.14 | 1354 | 1121 | 17.21 | 19.4 |
| Jilv3 | 112 | 105 | 6.25 | 255 | 231 | 9.41 | 1755 | 1589 | 9.46 | 8.3 |
| Pacific Early | 108 | 70 | 35.19 | 241 | 136 | 43.57 | 1689 | 915 | 45.83 | 83.2 |
| JWC1 | 89 | 62 | 30.34 | 149 | 101 | 32.21 | 1257 | 897 | 28.64 | 33.3 |
| Jin guan | 95 | 70 | 26.32 | 204 | 136 | 33.33 | 1687 | 1244 | 26.26 | 41.7 |
| Patron | 124 | 86 | 30.65 | 275 | 201 | 26.91 | 1984 | 1574 | 20.67 | 30.6 |
| Jing gang701 | 104 | 79 | 24.04 | 241 | 191 | 20.75 | 1782 | 1402 | 21.32 | 22.2 |
| Champion | 97 | 65 | 32.99 | 217 | 162 | 25.35 | 1854 | 1435 | 22.60 | 58.3 |

**Procedures of pre-evaluations of the drought-resistance:**

To estimate the drought-resistance,18 asparagus cultivars were grown in plastic pots (20 cm in diameter; 20cm in depth) with soil layers of 15 cm depth in 2019 at Dahe Experimental Station of Hebei academy of agriculture and forestry sciences (38.11 N, 114.38 E). Each cultivar grew in 18 pots and each pot contained 1 asparagus seedling. Equal amount of water was used in the experiment for those pots until the drought treatment. Half of the 18 pots were treated by drought and the other half remained well-watered as controls. We maintained the soil water content (SWC) in the drought treatment group at 45–50%. We maintained SWC in the control group at 75–80%. The drought treatment was started from the 71st day after sowing and lasts for 15 days. 4 traits were determined, including plant height (PH), the dry weight of plant, the fresh weight of plant, and the drought-injury index (DI).

Ranking of the standard and calculation of the drought-injury index (DI) fol-lowed the method of Zhang et al. [1], with some modifi-cation. The ranking criteria of drought-injury are presented in Table 1. DI was calculated using the following equation.

DI=[∑(N_0_×0+N_1_×1+ N_2_×2+ N_3_×3+ N_4_×4)/ N×4]×100%

Table 1 The ranking criteria of seedlings after salt treatment.

| Level | Ranking criteria |
| --- | --- |
| 0 | Normal growth without symptoms of injury |
| 1 | Approximately normal growth but with chlorosis and etiolation symptoms at the top of newborn spears |
| 2 | Inhibited growth and with etiolated leaves (cladodes) in all newborn spears and less than 20% dried stalks |
| 3 | Severely inhibited growth and with completely etiolated leaves aboveground and between 20% and 60% dried stalks |
| 4 | Almost dead or certified death and with more than 60% dried stalks |

**Reference**

[1] Zhang C, Hong B, Li J, Gao J. A simple method to evaluate the drought tolerance of ground-cover chrysanthemum (dentranthema×grandiflorum) rooted cuttings. Sci Agricultura Sinica. 2005; 38:789-96. (in Chinese)
